# The November-2023–March-2024 malaria epidemic in Zanzibar: a spatiotemporal epidemiological analysis

**DOI:** 10.1186/s12936-025-05507-2

**Published:** 2025-10-22

**Authors:** Frederike Kooiman, Mohamed Haji Ali, Michael Alifrangis, Shija Joseph Shija, Wahida Shirazi Hassan, Karin Linda Schiøler, Fatma Saleh

**Affiliations:** 1 School of Global Health, University of Copenhagen, Copenhagen, Denmark; 2 Department of Global Health, Amsterdam Institute for Global Health and Development, Amsterdam UMC, Location University of Amsterdam, Amsterdam, The Netherlands; 3 Zanzibar Malaria Elimination Programme (ZAMEP), Zanzibar, Tanzania; 4 Centre for Translational Medicine and Parasitology, Department of Immunology and Microbiology, University of Copenhagen, Copenhagen, Denmark; 5 Department of Infectious Diseases, Copenhagen University Hospital, Copenhagen, Denmark; 6 Global Health Section, University of Copenhagen, Copenhagen, Denmark; 7 School of Health and Medical Sciences, The State University of Zanzibar, School, Zanzibar, Tanzania

**Keywords:** Malaria, Epidemiology, Risk factors, Disease outbreaks, Disease hotspot, Weather, Rain, Temperature, Tanzania

## Abstract

**Background:**

In Zanzibar, malaria epidemiology has changed within the past 10 years, from focal, near-elimination transmission to recurrent and more widespread outbreaks. The observed changes culminated in a large-scale epidemic between November 2023 and March 2024 including 23,569 confirmed malaria cases.

**Methods:**

This study investigated the epidemiological characteristics of the 21-week outbreak by characterizing the risk profile of affected individuals, identifying malaria hotspots across space and time, and determining the association between malaria incidence and precipitation and temperature.

**Results:**

Males, individuals aged 15–35, urban residents, and those reporting to not sleep under insecticide-treated nets had a higher malaria risk. One significant space–time cluster was identified in the urban southwest of Unguja. The weekly number of malaria cases was significantly associated with the average weekly temperature, with an 8-week lag time.

**Conclusions:**

The results indicate a serious setback in the pursuit of malaria elimination in Zanzibar and call for intensified malaria interventions targeting high-risk populations.

**Supplementary Information:**

The online version contains supplementary material available at 10.1186/s12936-025-05507-2.

## Background

Zanzibar has historically shown impressive results in the pursuit of malaria elimination. A remarkable 96% reduction in the community prevalence of *Plasmodium falciparum* was observed in Zanzibar from 2003 to 2015 [1]. Unfortunately, the goal of malaria elimination has suffered serious setbacks in recent years. Notably, the annual parasite prevalence (API) increased from 2.7 cases per 1000 people in 2017 to 3.6 cases per 1000 people in 2021 [2]. Moreover, recurrent outbreaks have been reported, including an epidemic in 2019–2020, an outbreak in May–July 2023 with 1993 confirmed cases, and most recently, a massive epidemic from November 2023–March 2024, including 23,569 confirmed cases [2–5].

Malaria control in Zanzibar is impeded by multiple factors, all of which may have contributed to the increase in cases since 2017 and the 2023–2024 epidemic. First, climate change makes outbreaks more unpredictable through alterations in malaria dynamics such as larval mosquito development rates, parasite development (sporogony) rates, mosquito survival and biting behaviour and the availability of larval mosquito development sites [6–9].

In addition, importation of malaria from neighbouring regions with higher malaria incidence is thought to impede malaria elimination in Zanzibar, especially due to the high frequency of travel to and from mainland Tanzania [2, 10–13]

Furthermore, malaria elimination efforts are hampered by the declining effectiveness of key malaria-control interventions [2, 14]. In Zanzibar, this is believed to be the result of mosquito behaviour change of *Anopheles arabiensis* and insecticide resistance rendering long-lasting insecticidal nets (LLINs) and indoor residual spraying (IRS) less effective [2, 14].

The profound changes in malaria transmission in Zanzibar, call for reassessment of current malaria control strategies. This comprehensive epidemiological study aimed to inform future malaria interventions by investigating the epidemiological characteristics of the 2023–2024 malaria outbreak in Zanzibar. More specifically, this study assessed the risk profile of affected individuals, identified malaria hotspots across space and time, and examined the correlation between malaria transmission, precipitation and temperature.

## Methods

### Study site

Zanzibar, an archipelago off Tanzania, consists of two main islands, Unguja and Pemba. Zanzibar’s population is approximately 1.9 million and it is divided into 11 districts and 388 Shehias, the smallest administrative unit [15, 16]. Between the two main islands, Unguja is larger, more densely populated, and more urban [16]. It is a popular tourist destination with frequent travel to and from its port and airport in Mjini (Zanzibar City) [16]. Pemba’s population is younger and mostly employed in the agricultural sector, and experiences higher poverty rates [17]. Malaria transmission generally intensifies during the two rainy seasons, usually peaking after the main rainy season in March–May (*Masika*) [18]. The primary malaria vector in Zanzibar is *An. arabiensis*, followed by *Anopheles merus*, both belonging to the *Anopheles gambiae* complex [19]. The main malaria parasites are *P. falciparum*, accounting for about two-thirds of cases, followed by *Plasmodium malariae* (26.2%), and 6.6% of cases were caused by mixed infections [20]. Diagnosis is obtained via rapid diagnostic tests (RDTs) and if laboratory services are available the diagnosis is confirmed via microscopy [2]. All public health facilities provide malaria diagnosis and treatment free of cost [2].

### The disease surveillance and response system of Zanzibar

Zanzibar’s malaria control programme relies heavily on artemisinin-based combination therapy (ACT), introduced in 2003/2004, combined with indoor residual spraying (IRS), long-lasting insecticidal nets (LLINs) and rapid diagnostic tests (RDTs) [1]. Insecticide-treated net (ITN) usage rates differ between Unguja and Pemba: about half of Unguja residents reported sleeping under an ITN the previous night compared to 72.8% of Pemba residents [21]. Furthermore, ITN usage was higher among children under 5 and pregnant women [21]. IRS is implemented in targeted Shehias after reaching a threshold of three local cases per week and entomological investigation [2]. From January to December 2024, 52 out of 129 Shehias (40.3%) on Pemba received at least one round of IRS and on Unguja, 75 out of 259 Shehias (29.0%) received IRS (B. Khatib, pers. comm.).

Zanzibar’s malaria surveillance system encompasses the integrated disease surveillance and response (IDSR) strategy since 2010 and the Malaria Case Notification (MCN) system since 2011 [22–24]. The Malaria Early Epidemic Detection System (MEEDS) was used in parallel to the IDSR and MCN system until 2022, when it was discontinued [23].

Reactive Case Detection (RACD) was introduced in 2012, and Active Case Detection in 2016 [20, 25]. The MCN system incorporates RACD through the deployment of District Malaria Surveillance Officers (DMSOs) who follow up on cases and test their household members [20]. Upon confirmation of a malaria diagnosis at a health facility, a case must be documented into the National Malaria Case Register (MCR) by SMS [20]. DMSOs aim to visit health facilities within 24 h after detection of a new case to verify it and collect additional information about the individual [20]. The case is then submitted to the MCN system. Subsequently, DMSOs plan a visit to the household to gather extra data from the confirmed malaria case, collect information about household members, and test them for malaria [20]. Malaria medications, prescriptions for future use, and LLIN coupons are provided when needed [20]. Initially, the MCN system was only operational in public health facilities, but it was expanded to private health facilities in 2015 [25].

Besides the MCN system, the Ministry of Health began efforts to implement an eIDSR system in 2019 and introduced the DHIS2 platform to manage the data [23]. The eIDSR system collects weekly health-facility-based reports, including the number of suspected and confirmed malaria cases and the number of individuals tested for malaria [23]. While DHIS2 only contains cases that were tested at a facility, the MCN system also reports cases identified through RACD, including asymptomatic cases and cases who did not seek care at a facility. In addition, reporting to the MCN system is more urgent, as it must be completed within 24 h whereas reporting to DHIS2 occurs weekly, potentially making the latter more prone to underreporting. MCN data is therefore believed to represent the true number of cases more accurately.

ZAMEP performs data analysis weekly to detect exceeded epidemic thresholds, assess the time to treatment, the percentage of cases of severe malaria, the epidemic curve, and the case fatality rate [20, 26]. In addition, the Surveillance Monitoring and Evaluation (SME) team reviews the data to assess whether the surveillance guidelines are being adhered to and whether the data quality and timeliness are up to standard [27].

### Ethical considerations

This study was approved by the Zanzibar Health Research Ethics Review Committee (ZAHREC) of the Zanzibar Health Research Institute (ZAHRI) and a research permit was obtained from the Research Council at the Second Vice President’s office (Ref.: 2,001,716,138,240,306,222,829). The Zanzibar Malaria Elimination Programme (ZAMEP) granted access to the malaria surveillance data and obtained informed consent with respect to patient and other personal data registration in the national malaria surveillance systems.

### Study design and data analysis

This retrospective, longitudinal study analysed malaria surveillance data collected from January 2023 to March 2024, stored in Zanzibar’s Health Management Information Systems: District Health Information Software 2 (DHIS2) and the Malaria Case Notification (MCN) system. The data, which included personally identifiable information, were accessed solely by the first author on the 22nd of May 2024. Additionally, the study used publicly available population data and meteorological data. The quantitative data used and their sources are listed in S1 Table. Data analysis was conducted at the ZAMEP head office in Unguja, enabling communication with surveillance officers who provided clarifications to support data cleaning. Preliminary findings were presented to ZAMEP's management, whose input was used to align the study with their needs and resulted in an additional analysis.

### Outcome measures

First, incidence rates (IRs) and test positivity rates (TPRs) were calculated as the number of malaria cases per 1000 people at risk per unit of time and the proportion of tests that returned a positive malaria result, respectively. Population size data for the year 2023 were used as the denominator when calculating the incidence rates for Zanzibar, Unguja and Pemba [15]. The IR and TPR were calculated over three time periods: (i) Jan. 2023–March 2024 (full study period); (ii) Jan. 2023–Dec. 2023 (2023 API); and (iii) week 13, 2023–week 13, 2024. The latter period captured the full epidemic while maintaining comparability with other yearly incidence rates. The IRs were calculated using the weekly number of cases reported in MCN. The TPRs for Zanzibar for each period were calculated using DHIS2 data based on the availability of data on the number of RDTs performed weekly. IRs were calculated for each Shehia. The Shehia population sizes for 2023, used as the denominator in IR calculations, were estimated by taking the 2022 population sizes [15, 28], and adjusting for population growth. The specific growth rates for urban (4.3%), rural (3.2%), and mixed (3.7%, average growth rate) were applied [15].

The weekly numbers of imported and indigenous cases were presented temporally. Cases were classified as indigenous in the MCN system when there was no self-reported history of overnight travel outside Zanzibar 30 days prior, according to ZAMEP’s definition. Cases that did report such a travel history were classified as imported.

The occupations of confirmed malaria cases who were followed up at the household are presented, stratified by sex.

### Risk and risk ratios

Risk was calculated as the cumulative number of confirmed malaria cases from January 2023 to March 2024 within a given risk group, divided by the total number of individuals in that risk group in the general population, based on 2023 census data [15, 21, 28]. Risk ratios (RRs) and their 95% confidence intervals (CIs) were calculated for sex; age; status of Shehia of residence (urban, rural or semi-urban); and insecticide-treated net (ITN) use (previous night), given the availability of risk factor data in the malaria surveillance data. The risk profile of the affected population was generated using MCN data, as it included individual risk factor data from confirmed malaria cases. The risk ratios and their 95% CIs were computed using the Wald method in the ‘epitools’ package in R Statistical Software (v4.4.0).

The results were stratified by sex and Shehia status to control for confounding by these factors, which were believed to be among the most influential. Shehia population sizes were adjusted for population growth since 2022 using specific rates for urban (4.3%), rural (3.2%), and semi-urban (3.7%, average growth rate) areas [15].

### Spatiotemporal cluster analysis

The spatiotemporal distribution of malaria cases per Shehia was examined via space–time cluster analysis using SaTScan™ [29]. This analysis applied the Poisson-probability model and ensured sufficient power to define statistically significant clusters by conducting 999 Monte Carlo replications [29]. Risk factors identified in the prior analysis were included as covariates to be adjusted for in the cluster analysis. Microsoft Excel, IBM SPSS Statistics (v28.0.2), R Statistical Software (v4.4.0) and QGIS (v3.36.0) were used for data analysis and visualization [30–32].

### Correlation with meteorological factors

A cross-correlation analysis was conducted to assess the relationships between temperature, rainfall and malaria cases, and to identify lag times. This analysis was performed on the weekly case counts obtained from MCN. Weekly average temperatures and total rainfall were obtained by aggregating hourly temperature data from ECMWF Reanalysis v5 (ERA5) [33] and daily Climate Hazards Group InfraRed Precipitation with Station data (CHIRPS) [34], downloaded from https://climateserv.servirglobal.net/. The cross correlation was assessed using the cross-correlation function (CCF) in R Statistical Software (v4.4.0).

## Results

### Malaria incidence

**Fig. 1:**
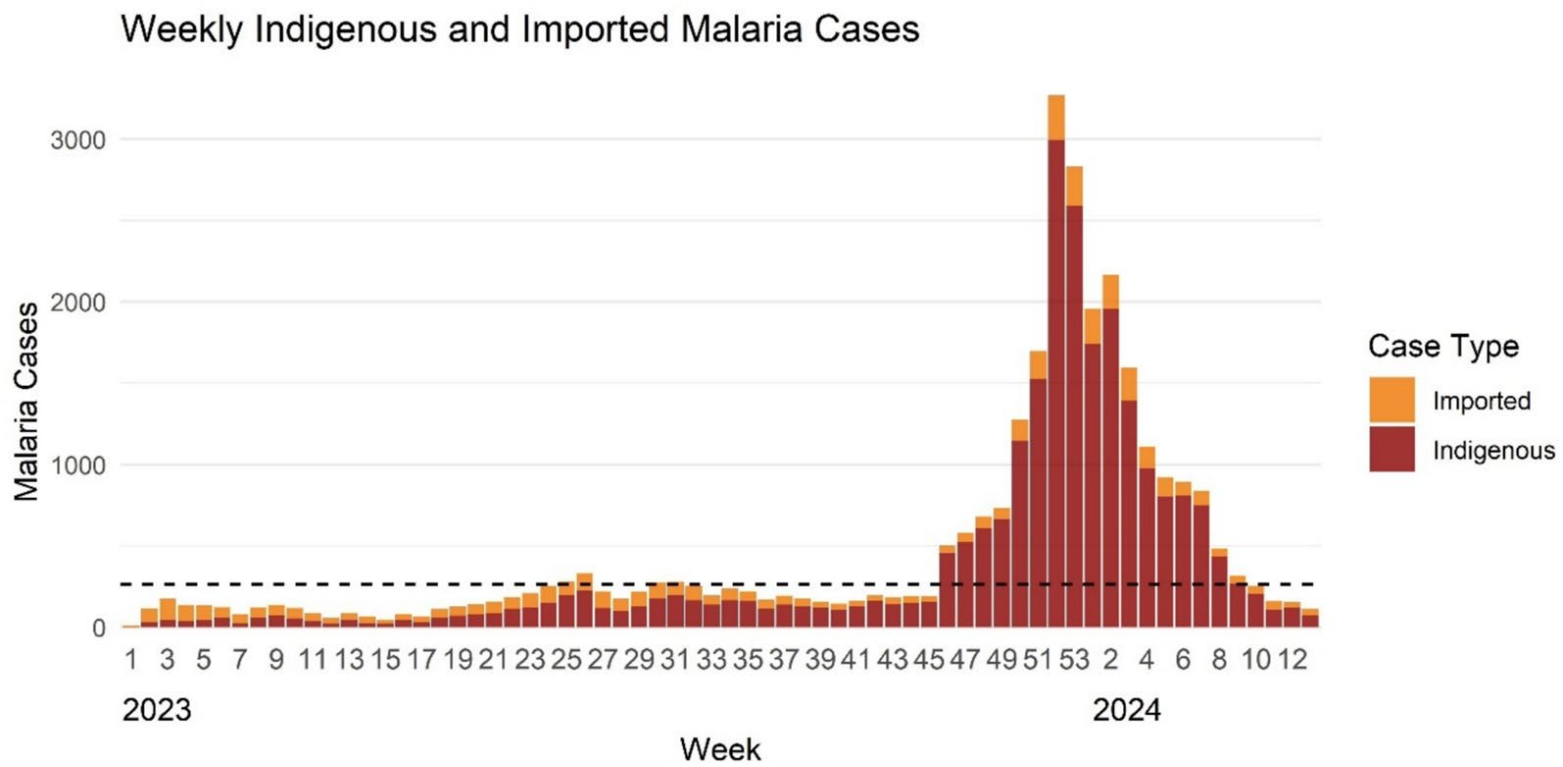
Epi-curve of confirmed malaria cases by week from Jan. 2023 to March 2024, comprising indigenous and imported cases. The dashed line indicates the epidemic threshold for malaria in Zanzibar (264 cases per week) [5]

From January 2023 to March 2024, a total of 30,044 confirmed malaria cases were reported in Zanzibar, resulting in an IR of 15.3 cases per 1,000 people and a TPR of 4.4% (Table 1). The epi-curve in Fig. 1 illustrates the weekly number of confirmed malaria cases recorded by MCN, comprising both imported and indigenous cases. The dashed line represents the epidemic threshold for Zanzibar, defined as 264 cases per week [5]. A total of 21,860 malaria cases were confirmed during the outbreak period of 17 weeks, with observed onset in week 46 of 2023. The outbreak peaked in week 52 with 3270 confirmed cases (Fig. 1).

**Table 1:** Malaria incidence, Annual Parasite Incidence (API) and Test Positivity Rate (TPR) in Zanzibar across three different time periods

		Zanzibar	Unguja	Pemba
1 Jan 2023–31 Mar 2024	Cases	30,044	28,897	1,147
	Incidence	15.3	20.7	2.0
	TPR	4.4%	NA	NA
Week 13, 2023–Week 13, 2024	Cases	29,712	28,658	1,054
	Incidence	15.2	20.5	1.8
	TPR	4.8%	NA	NA
1 Jan 2023–31 Dec 2023	Cases	19,011	18,359	652
	API	9.7	13.1	1.1
	TPR	3.7%	NA	NA

TPR, Test Positivity Rate; API, Annual Parasite Incidence; NA, not available

API and incidence are expressed as cases per 1000 population over the specified period

The incidence and number of cases were based on the weekly cases reported the Malaria Case Notification system (MCN)

From week 13, 2023 to week 13, 2024, there were 29,712 confirmed cases, resulting in an IR of 15.2 cases per 1000 people, and a TPR of 4.8%. The API for all of 2023 was 9.7 cases per 1000 people, with 19,011 confirmed cases and a TPR of 3.7% (Table 1). Notably, during the outbreak period between week 44 of 2023 and week 13 of 2024, the TPR rose to 7.5%.

Most cases (96.2%) were reported on Unguja, with only 3.8% reported on Pemba. On Unguja, 28,897 cases were reported from January 2023 to March 2024, with an IR of 20.7 cases per 1000 people (Table 1). On Pemba, 1147 cases were reported during this period, with an IR of just 2.0 cases per 1000 people. From week 13, 2023, to week 13, 2024, the IR was 20.5 on Unguja and 1.8 on Pemba. The 2023 API was 13.1 per 1000 population on Unguja and 1.1 on Pemba.

### Case classification

Most cases (82.6%) were indigenous, while 17.4% were imported (Table 2). However, the overall proportion of imported cases decreased from 39.1% before the epidemic, to 10.3% during the epidemic (Fig. 1).

**Table 2:** Travel history and case classification of confirmed malaria cases in Zanzibar from Jan. 2023 to March 2024

Characteristic	Zanzibar		Unguja		Pemba	
	Cases	%	Cases	%	Cases	%
**Case classification**						
Indigenous	24,821	82.6	24,218	83.8	603	52.6
Imported	5223	17.4	4679	16.2	544	47.4
**Travelled overnight in the past month**						
No	23,218	77.3	22,908	79.3	310	27.0
Yes, outside Zanzibar	4628	15.4	4142	15.6	486	42.3
Yes, within Zanzibar only	1663	5.5	1322	4.6	341	29.7
NA	535	1.8	525	1.8	10	0.9
Total	30,044	100	28,897	100	1147	100

Source: MCN. Case totals in this table represent MCN data only. Indigenous cases were defined as confirmed malaria cases with no self-reported history of overnight travel outside Zanzibar 30 days prior. Cases were classified as imported when individuals did report overnight travel outside Zanzibar within the past 30 days

### Occupations

Among individuals who tested positive for malaria and were followed up at their household (16,877 cases), a large proportion (25.8%) had missing data regarding their occupation (Table 3). This issue was slightly more prominent among males, with 27% of cases lacking occupation data, compared to 22% in females. For those with recorded occupational data, ‘Business’ was the largest category (17.1%), followed by ‘student’ (14.3%) and ‘other’ (12.6%). The occupations of confirmed malaria cases were different in females and males, with the most common occupation in women being ‘housewife’, whereas in men, most were categorized as ‘business’.

**Table 3:** Occupations of confirmed malaria cases in Zanzibar from Jan. 2023 to March 2024 by sex

Occupation	All		Male		Female	
	Cases	%	Cases	%	Cases	%
Business	2884	17.1	2437	19.7	447	10.0
Civil servant	249	1.5	212	1.7	37	0.8
Farmer	733	4.3	588	4.7	145	3.2
Fisherman	664	3.9	652	5.3	12	0.3
Health worker	26	0.2	14	0.1	12	0.3
Housewife	1027	6.1	60	0.5	967	21.6
Student	2409	14.3	1551	12.5	858	19.2
Watchman/Police	1021	6.0	992	8.0	29	0.6
Unemployed	766	4.5	581	4.7	185	4.1
Under school age (< 6)	632	3.7	340	2.7	292	6.5
Other	2119	12.6	1,619	13.1	500	11.2
Missing	4347	25.8	3,339	27.0	984	22.0
Total	16,877	100	12,385	100	4468	100

Source: MCN household follow-up data

### Spatial distribution of malaria cases across Zanzibar

Out of 30,044 cases reported across Zanzibar in the MCN system, 29,980 could be matched with a Shehia ID number. These cases were subsequently presented on incidence maps, their Shehia status was determined, and they were included in the cluster analysis.

**Fig. 2:**
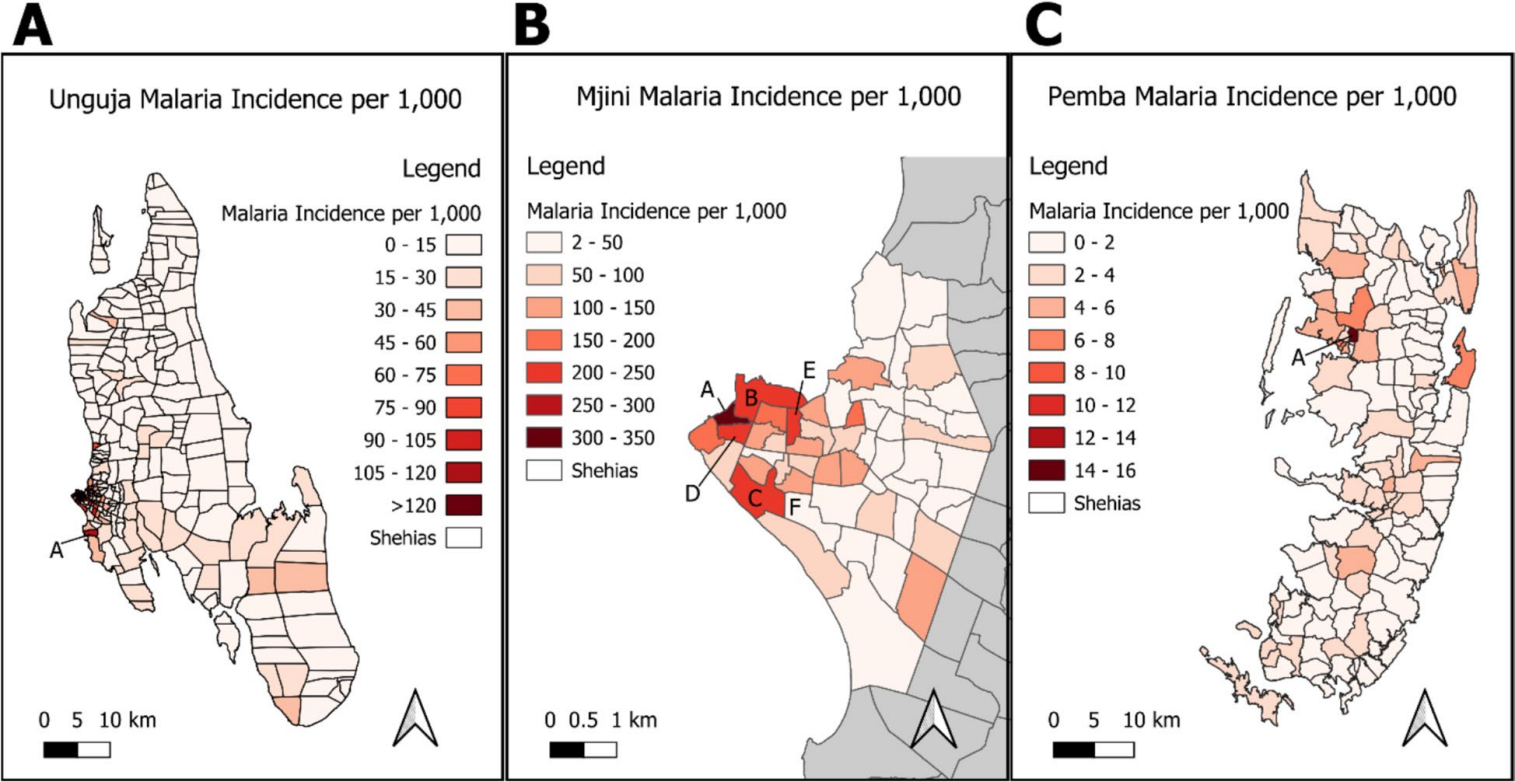
Malaria incidence per 1000 population from Jan. 2023 to March 2024 on Unguja (**A**) (N = 28,897), in Mjini (B) (N = 12,953), and on Pemba (**C**) (N = 1147)

The IR over the whole period from January 2023 to the end of March 2024 is depicted in Fig. 2 with separate maps for Unguja (2A), Mjini (home to Zanzibar City, the regional capital) (2B), and Pemba (2C). On Unguja overall, from January 2023 to March 2024, the highest incidences, up to 344 cases per 1,000 population, were observed in Shehias in Stone Town within Mjini district (Table 4 and Fig. 2B). When excluding Mjini district, the highest incidence was observed in Kiembesamaki Shehia with 116 cases per 1000 (A, Fig. 2A). On Pemba, the malaria incidence per Shehia ranged from 0 to 14.7 per 1000 population in Kizimbani (A, Fig. 2C).

Incidence maps for April (week 13) 2023 to April (week 13) 2024 and the 2023 API are presented in the S1 and S2 Figs to enable comparisons with incidence maps reported elsewhere.

**Fig. 3:**
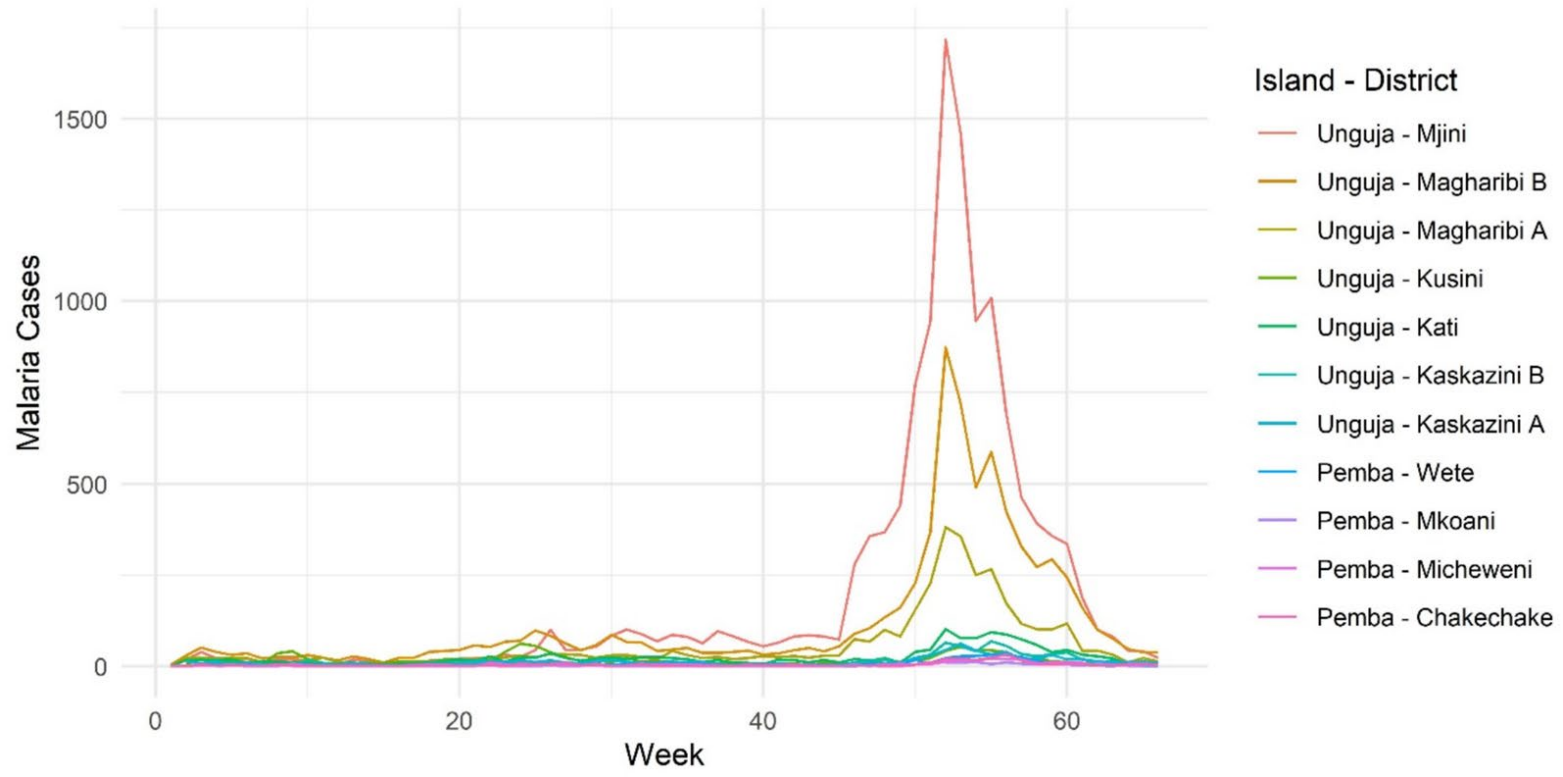
Weekly malaria cases per district from Jan. 2023 to March 2024

Figure 3 demonstrates that the start of the epidemic from week 46 was mainly concentrated in Mjini and subsequently spread to Magharibi A and Magharibi B, whereas the cases during the surge around week 25 were more evenly distributed across the same three districts in Unguja.

**Table 4:** Zanzibar Shehias with the highest malaria incidence per 1000 population from Jan. 2023 to March 2024

Shehia	Incidence (cases per 1000 population)
Kiponda (A, Fig. 3B)	344
Malindi (B, Fig. 3B)	242
Kikwajuni Juu (C, Fig. 3B)	228
Mlandege (D, Fig. 3B)	209
Mkunazini (E, Fig. 3B)	202

### Malaria risk ratios for sex, Shehia status, and ITN use

Malaria risk factors were identified by calculating risk ratios (RRs). The malaria risk factor data are presented in Table 5, along with general population data for comparison, and risks and RRs. More than three-quarters (76.9%) of the confirmed cases were male, with a risk of 2.4% compared to 0.7% for females (Table 5). Men, therefore, had a 3.5 times greater risk of malaria than women (RR = 3.5; 95% confidence interval 3.5–3.6). During the start of the epidemic in weeks 46–50, an even higher proportion of cases was male (81.0%), indicating that the epidemic started in this part of the population.

**Table 5:** Descriptive statistics and risk ratios for confirmed malaria cases from Jan. 2023 to March 2024 in Zanzibar

Risk factor	Cases	%	Pop. 2023	%	Risk (%)	RR (95% CI)
Total	30,044	100	1,959,365	100	NA	NA
**Sex, n = 30,044**						
F	6,938	23.1	1,010,330	51.6	0.7	1
M	23,106	76.9	949,365	48.4	2.4	3.54 (3.45–3.64)
**Age group, n = 30,044**						
< 5	1,069	3.6	286,115	14.6	0.4	1
5–15	3,117	10.4	513,440	26.2	0.6	1.63 (1.52–1.74)
15–25	10,355	34.5	397,818	20.3	2.6	7.0 (6.5–7.4)
25–35	7,563	25.2	299,833	15.3	2.5	6.8 (6.3–7.2)
35–45	3,693	12.3	195,970	10.0	1.9	5.0 (4.7–5.4)
45 +	4,247	14.1	266,519	13.6	1.6	4.3 (4.0–4.6)
**Shehia status, n = 30,044**						
Rural	3,827	12.7	744,383	37.9	0.5	1
Semi-urban	3,875	12.9	409,768	20.9	0.9	1.84 (1.76–1.92)
Urban	22,278	74.2	809,331	41.2	2.8	5.35 (5.17–5.54)
Missing	64	0.2				
**ITN use previous night, n = 16,877**						
Yes	4,970	29.4	1,117,027	57	0.4	1
No	7,549	44.7	842,669	43	0.9	2.01 (1.94–2.09)
Missing	4,358	25.8				

Children under 5 years were the age group with the lowest risk, at just 0.4% (Table 5). Using this age group as a reference, the highest RR was observed in persons aged 15–25 years (RR = 7.0; 6.5–7.4). Similarly, the risk of malaria in individuals aged 25–35 years was 6.8 times higher than in those under 5 years of age (RR = 6.8; 6.3–7.2). The RRs for the 35–45 years and 45 + years age groups were 5.0 (4.7–5.4) and 4.3 (4.0–4.6), respectively. Lastly, the risk of malaria in children aged 5–15 years was 1.63 times greater than in those under 5 years (RR = 1.63; 1.52–1.74).

The risk of malaria was more than five times greater for people living in urban Shehias (2.8%) than for those in rural Shehias (0.5%)(RR = 5.35; 5.17–5.54) (Table 5). Notably, the risk of malaria in semi-urban Shehias was nearly twice that of rural Shehias (RR = 1.84; 1.76–1.92).

Lastly, malaria risk was twice as high for individuals who did not sleep under an ITN the previous night (RR = 2.01; 1.94–2.09) (Table 5). In children under 5 years old, the risk of malaria was 1.38 times greater for those who did not use an ITN (RR = 1.38; 1.18–1.61) (S2 Table). The total case counts for ITN use are inconsistent with the total number of cases reported in the MCN system (30,044) because only 16,877 of those cases were followed up at the household to collect these data.

### Malaria risk ratios stratified by sex and Shehia status

When the data were stratified by sex (Table 6), age was found to have a stronger effect on malaria risk in males than in females. Among males, the highest RR was observed in the 25–35 age group (RR = 10.4; 9.6–11.4). In females, however, the highest RR was observed in the 15–25 age group (RR = 3.26; 2.95–3.61).

**Table 6:** Malaria risk ratios stratified by sex in Zanzibar from Jan. 2023 to March 2024

Risk factor	Male			Female		
	Risk (%)	RR	(95% CI)	Risk (%)	RR	(95% CI)
**Age group,** **n = 30,044**						
< 5	0.4	1		0.3	1	
5–15	0.7	1.71	(1.56–1.88)	0.5	1.51	(1.36–1.68)
15–25	4.3	10.2	(9.4–11.0)	1.1	3.26	(2.95–3.61)
25–35	4.4	10.4	(9.6–11.4)	0.8	2.56	(2.30–2.84)
35–45	3.2	7.6	(7.0–8.3)	0.7	2.23	(1.98–2.50)
45 +	2.5	6.0	(5.5–6.5)	0.7	2.14	(1.92–2.39)
**Shehia status, n = 30,044**						
Rural	0.5	1		0.2	1	
Semi-urban	1.9	3.41	(3.24–3.60)	0.6	2.94	(2.70–3.20)
Urban	5.7	10.5	(10.1–10.9)	1.5	6.73	(6.31–7.18)

RR, risk ratio. The number of decimal places used to report risk ratios was based on the rule of four [35]. Source: MCN; [15, 28]

Similarly, Shehia status had a stronger effect on malaria risk in males than females. Males living in urban Shehias had a 10.5 times higher risk of malaria than those in rural Shehias (RR = 10.5; 10.1–10.9), whereas females living in urban Shehias had a 6.73 times higher risk compared to those in rural Shehias (RR = 6.73; 6.31–7.18) (Table 6).

Stratification by Shehia status revealed that age plays a more pronounced role in malaria risk in semi-urban and urban Shehias than rural Shehias, with larger RRs observed in these areas (Table 7). In semi-urban Shehias, the age group 25–35 was most at risk (4.0%), whereas in urban Shehias, individuals aged 15–25 were most at risk (5.9%), followed by those aged 25–35 (5.4%). Additionally, in urban Shehias, males had a 3.94 times higher risk than females (RR = 3.94; 3.81–4.06) whereas in semi-urban and rural Shehias, males had a 2.52 times higher risk (RR = 2.52; 2.35–2.71, and RR = 2.52; 2.35–2.70, respectively).

**Table 7:** Malaria risk ratios stratified by status of Shehia of residence in Zanzibar from Jan. 2023 to March 2024

Risk factor	Rural		Semi-Urban		Urban	
	Risk (%)	RR (95% CI)	Risk (%)	RR (95% CI)	Risk (%)	RR (95% CI)
**Sex,** **n = 30,044**						
F	0.2	1	0.7	1	1.5	1
M	0.5	2.52 (2.35–2.70)	1.7	2.52 (2.35–2.71)	5.7	3.94 (3.81–4.06)
**Age,** **n = 30,044**						
< 5	0.2	1	0.5	1	0.7	1
5–15	0.2	1.00 (0.86–1.16)	0.5	0.97 (0.82–1.14)	1.5	2.23 (2.04–2.45)
15–25	0.6	3.55 (3.12–4.05)	2.0	4.36 (3.79–5.02)	5.9	8.9 (8.2–9.7)
25–35	0.6	3.54 (3.09–4.05)	4.0	8.6 (7.5–10.0)	5.4	8.2 (7.6–9.0)
35–45	0.4	2.46 (2.11–2.86)	1.8	3.92 (3.36–4.57)	4.1	6.2 (5.7–6.8)
45 +	0.3	1.78 (1.53–2.06)	1.1	2.43 (2.07–2.85)	3.6	5.5 (5.1–6.0)

The number of decimal places used to report risk ratios was based on the rule of four [35]. Source: MCN; [15, 28]

### Identifying malaria hotspots using cluster analysis

**Fig. 4:**
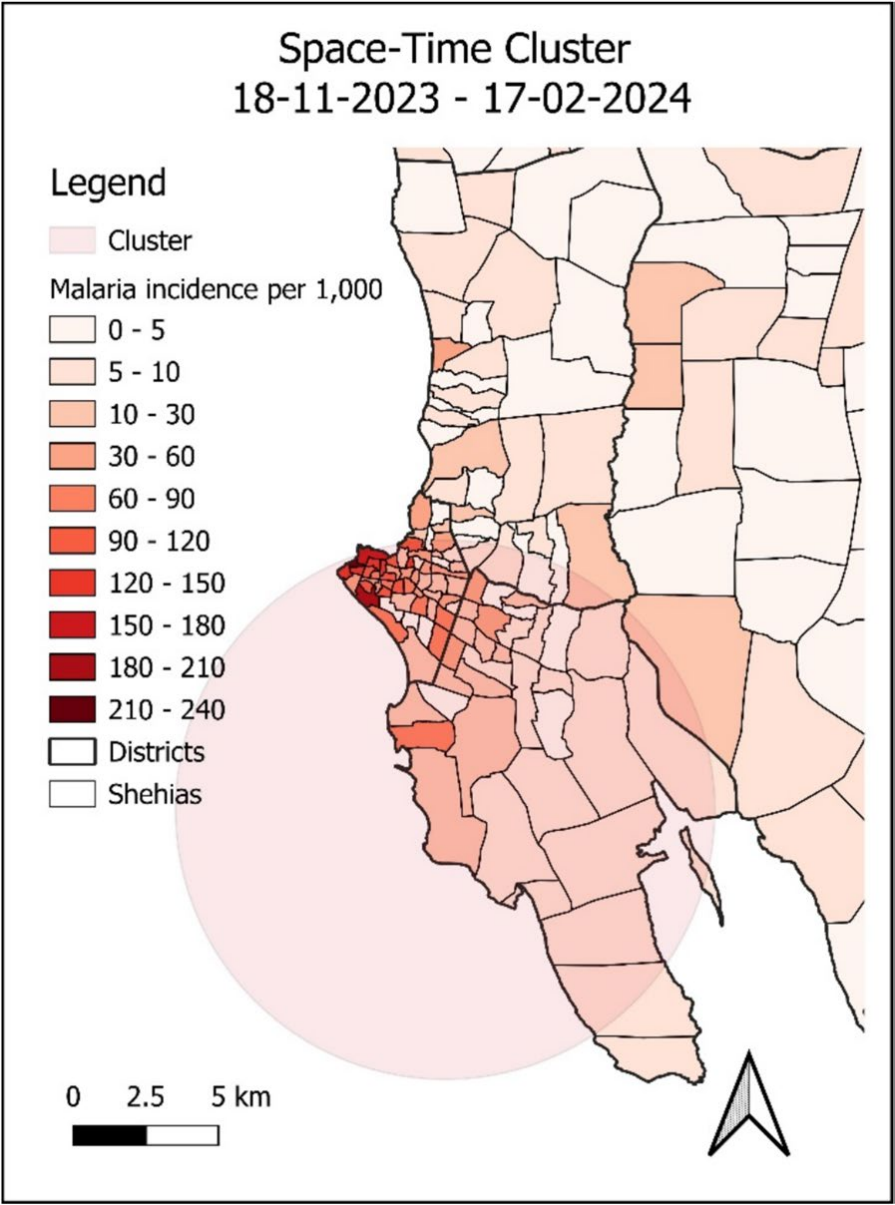
Space–time cluster and malaria incidence per 1000 population in southwest Unguja from the 18th of Nov. 2023 to the 17th of Feb. 2024. Source: MCN

A single space–time cluster was identified in the southwest of Unguja, covering most of the districts Mjini and Magharibi B from the 18th of November, 2023, to the 17th of February, 2024 (Fig. 4). According to the analysis, within this cluster, the time frame of the outbreak spanned from week 46 of 2023 to week 7 of 2024. After adjusting for the sex and age distribution, and the urban classification of each Shehia, the RR of malaria within this space–time cluster was 9.12 compared to outside the cluster with 14,847 recorded malaria cases against 2913 expected cases.

### The association between weather and malaria incidence

**Fig. 5:**
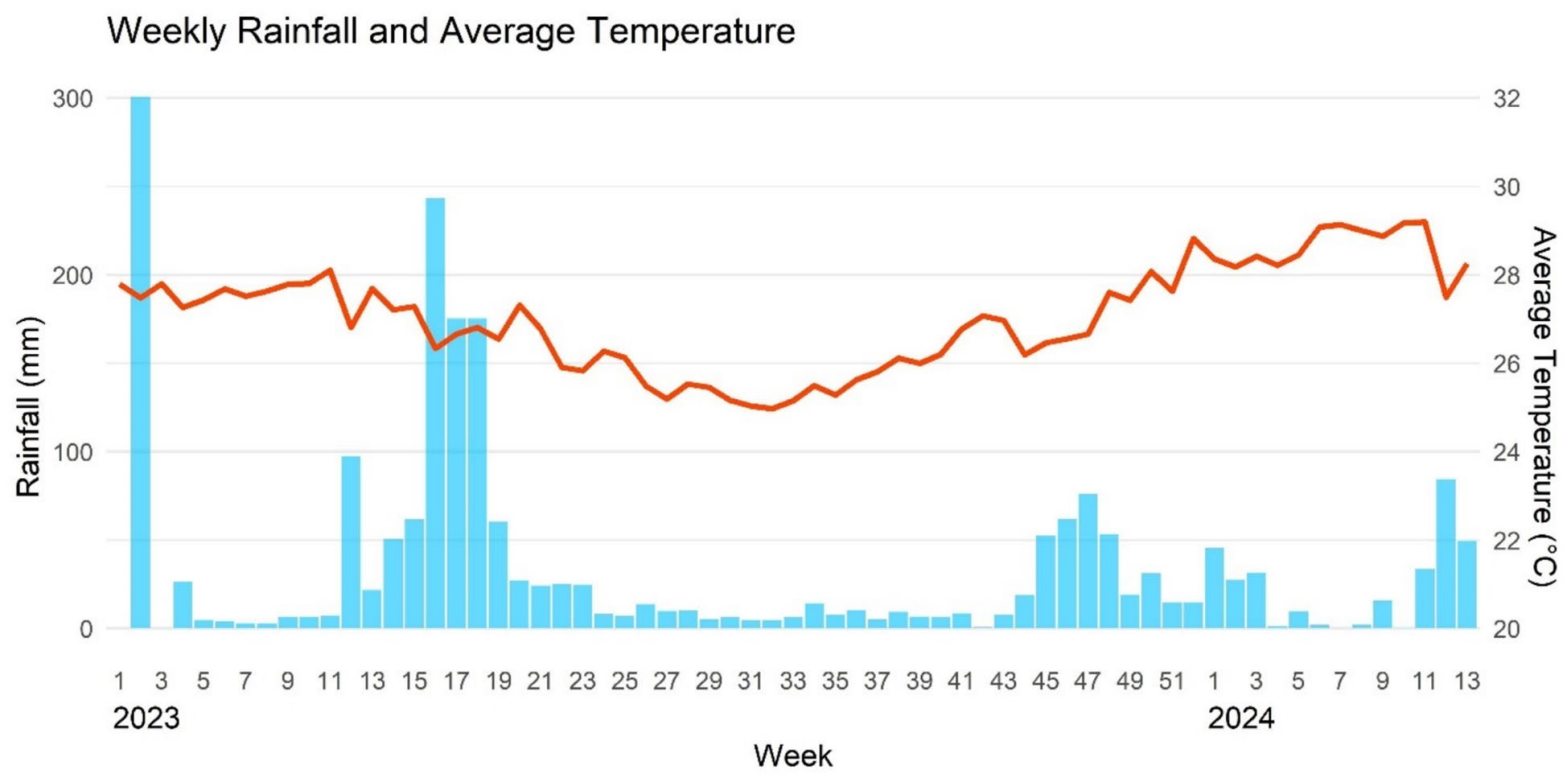
Weekly rainfall (mm) and average temperature (°C) in Zanzibar from Jan. 2023 to March 2024. Sources: [33, 34]

The weekly rainfall and average temperature during the study period are presented in Fig. 5. From January 2023 to March 2024, there was a total of 2146 mm of rain, with 1843 mm in 2023. The 2023 main rainy season (Masika) peaked in week 16 (mid-April) with 243 mm and the short rainy season (Vuli) peaked in week 47 with 76 mm. Week 2 of 2023 is an outlier with 300 mm of rain. Average weekly temperatures ranged from 25 °C in August to 29.2 °C in March 2024.

The cross-correlation analysis did not indicate a significant correlation between weekly total rainfall and malaria cases (S3A Fig). However, a significant (p < 0.05) positive correlation of moderate strength (Autocorrelation function (ACF) 0.616) was observed between weekly average temperature and malaria cases, with an 8-week lag (Fig. S3B).

## Discussion

This study revealed an unusually high malaria incidence in Zanzibar from January 2023 to March 2024, due to a large-scale epidemic with onset in November and peak in December of 2023. It follows only 3 years after another major epidemic. The 2023 API of 9.7 cases per 1000 people is considerably higher than the APIs recently reported for 2017 to 2021 [2]. Between 2017 and 2021, the API ranged from 2.7 to 4.2 cases per 1000 people when excluding the epidemic year of 2020 which was also unusually high (8.5) due to an epidemic [2]. The extent of the 2023–2024 epidemic becomes even more apparent when considering the incidence from week 13, 2023 to week 13, 2024, of 15.2 cases per 1000 population. The epidemics and increasing incidence since 2017 indicate a worrying trend of intensified malaria transmission in an archipelago previously on track to achieving malaria elimination.

This study found a lower proportion of imported cases (17.4%) than a previous study investigating data from 2017 to 2021 (68%), likely due to increased local transmission during epidemics [2]. However, the pre-epidemic proportion of imported cases (39.1%) was also lower than in most previous years [2], suggesting higher local transmission rates throughout the study period, which is a concerning development.

The results indicate that male sex, ages 15–35, living in an urban Shehia, and not sleeping under an ITN were associated with a higher malaria risk. Conversely, being female, under 5 years old, living in a rural Shehia, and using an ITN were associated with a lower malaria risk. Generally, children living in endemic areas are at higher risk of malaria compared to adults, as adults often have acquired immunity through previous exposure [36]. However, the results of this study demonstrate a much higher risk in those aged 15 to 35 than in children younger than 5. The lower risk observed in children under 5 in this study is also reported in other studies in Mainland Tanzania [37], and Zanzibar [2]. The high rate of ITN use in children under 5 years achieved over the years is believed to have shifted the relative risk of malaria to older age groups, driven by delayed naturally acquired immunity and the disproportionate reduction of risk in younger children [13, 37].

Interestingly, a comparison of this study with Zanzibar’s MCN data from 2012 to 2019 suggests a shift in malaria risk from females to males [13]. The transition of the malaria burden to (young) adult men has been documented across various malaria-eliminating countries and underscores the importance of behavioural and occupational factors associated with increased exposure to malaria vectors [38–40]. Calculating RRs for occupations was impossible due to data limitations such as broad occupational categories, missing data, and a lack of comparable data for the general population. Therefore, more detailed occupational data from cases is required to clarify the relationship between occupation and malaria risk in Zanzibar. However, previous qualitative research and anecdotal evidence from DMSOs indicate that in Zanzibar, jobs involving outdoor activities, especially during the evening or night (such as farming, fishing, security, construction, street vending, tourism, and bodaboda (motorbike taxi) driving) increase men’s exposure to malaria vectors [5, 10] (W. S. Hassan, Surveillance Officer at ZAMEP, pers. comm.).

*Anopheles arabiensis*, the main malaria vector in Zanzibar, has shifted to exophagic behaviour and early evening peak biting times [2, 14, 41]. This interplay between outdoor human and mosquito activity could have contributed to the higher relative risk in men aged 15 to 35 and in urban areas, because there is considerably more human outdoor evening and night-time activity in urban areas compared to rural areas [10]. An entomological study found that exclusively in Stone Town, *An. gambiae *sensu stricto (*s.s*.) was more abundant than *An. arabiensis* (62% vs. 31%) [41]. *Anopheles gambiae s.s* bites primarily indoors and at night, making ITNs and IRS highly effective against this vector [41]. The low utilization of ITNs and lack of IRS implementation in Stone Town until December 2023 in response to the epidemic are potential reasons for the relatively high abundance of *An. gambiae s.s* in this area [41]. Therefore, malaria control strategies in Stone Town should include intensification of both indoor and outdoor interventions, such as IRS, increased utilization of ITNs by men, environmental management and larviciding. While outdoor interventions are resource-intensive due to the need for repeated implementation, they could also reduce the risk of arboviral diseases like dengue and chikungunya, transmitted by container-breeding *Aedes aegypti*, an emerging threat in Zanzibar [42]. The World Health Organization (WHO) recommends integrated vector management of both Anopheline and Aedine vectors to optimize resource use [43].

Additionally, the elevated risk in urban Shehias may be related to lower immunity, importation, and vectors’ adaptation to urban environments. Generally, malaria risk is reported to be higher in rural than urban areas, leading to lower immunity levels in urban areas [44–47]. Additionally, the urban centre of Unguja receives many travellers from areas where malaria is endemic, potentially importing the disease to the island. Notably, the incidence in this study was highest near Unguja’s port, where ferries and other vessels arrive from the mainland. As a result, such urban areas are vulnerable to large epidemics, especially in elimination settings like Zanzibar, where incidence has declined over the past decades, lowering the overall immunity level [44].

Some malaria vectors, such as *An. gambiae *sensu lato (*s.l.*) have been found to adapt to the urban environment, using manmade habitats, and polluted water bodies such as sewage ponds as breeding sites [44, 48–52]. This includes *An. arabiensis* in Unguja, whose larvae were detected at different construction sites during the surge around week 25 in 2023 [5]. Moreover, insufficient sanitation and waste disposal were cited as reasons for the higher malaria risk observed in Mjini, Magharibi A and Magharibi B during the 2023–2024 epidemic [53].

In other countries, including in East Africa, the spread of *Anopheles stephensi*, an efficient urban malaria vector often resistant to insecticides, further contributes to the rise in urban malaria [54, 55]. Additionally, genetic changes in *P. falciparum* which lead to increases in false-negative rapid diagnostic test (RDT) results, have been detected in other East African countries, such as Ethiopia, but were absent in Zanzibar [56]. Other genetic changes have led to partial resistance to artemisinin in Tanzania, causing a rise in treatment failure rates [6].While the presence of these genetic changes and *An. stephensi* has not been demonstrated in Zanzibar (B. Khatib, pers. comm.), the threat of their introduction persists [2, 56]. This calls for close surveillance to detect any of these changes early, to be able to respond appropriately.

While the previously discussed factors may partially explain the higher malaria risk in urban areas, the cluster analysis adjusted for sex, age, and Shehia status, suggesting that additional factors must have contributed to the higher risk observed in Mjini and Magharibi B. Historically, the districts with the highest number of malaria cases in Unguja have also been Mjini, Magharibi B, and Magharibi A [5]. This indicates that risk distribution across districts has remained relatively unchanged in Unguja. However, comparison with a risk stratification map from 2021 reveals changes in malaria risk per Shehia over time [57]. Shehias in Zanzibar have been stratified into four malaria risk categories with corresponding intervention packages: high diffuse transmission, moderate focal transmission, low focal transmission, and pre-elimination [57]. Most Shehias with the highest incidence in Mjini in this study were similarly classified under the high diffuse transmission stratum according to their stratification. However, discrepancies are observed in both Unguja and Pemba, where some Shehias classified as high diffuse transmission had very low incidence rates, while others classified under the pre-elimination stratum had relatively higher incidence rates in the current study. The vast differences in malaria risk across Shehias, and the changes in malaria risk per Shehia over time, demonstrate the importance of continuing to regularly analyse the spatial distribution of risk. Further research is needed to explain the increased risk in hotspot areas, particularly in certain Shehias such as Kiponda. This should include analyses of entomological data, and occupational and behavioural factors to inform future interventions addressing the new landscape of high-risk populations. Entomological data including insecticide resistance data, is sparsely distributed across the archipelago, making it impossible to include in the spatiotemporal analysis of this study. Therefore, other study designs must be employed to study these additional factors.

Associations between malaria and rainfall and temperature have been established in many studies [58–61], including in Zanzibar [1, 9]. Therefore, this study's lack of a significant association between rainfall and malaria cases was unexpected. This could be explained by the short time frame and the use of an absolute rainfall variable, which are limitations of this study. Consequently, our data did not capture the abnormality of the weather conditions during the study period compared to previous years, brought on by the Indian Ocean Dipole phenomenon and one of the strongest El Niño events ever recorded [62, 63]. In 2023 in Zanzibar, there was 125–150% more rainfall than the long-term average, especially during the Vuli season, alongside above-average temperatures for most of the year [64]. The positive association between temperature and malaria incidence found in this study could be explained by the accelerated development rate of *An. arabiensis* with increasing temperatures between 18 °C and 32 °C [8]. Consistent with this study’s findings, another study found that in low-altitude regions of East Africa, temperature was a significant driver of the monthly malaria entomological inoculation rate seasonality, whereas monthly rainfall had insignificant contributions [61].

In Zanzibar, climate change is believed to increase rainfall from March to May [65] and lead to more frequent strong El Niño and La Niña events [66]. This may alter malaria transmission patterns, demanding more research on the relationships between climate change and malaria in the region. Future studies should use variables like the Standardized Precipitation Index to capture the deviation from the long-term average amount of rain, alongside absolute rainfall measures.

## Limitations

In addition to the limited ability to capture the effects of abnormal weather conditions during the study period, and the constraints in the occupational data that prevented calculation of RRs for this variable, this study had several other limitations.

First, data errors were observed for some variables. For example, 60 male cases had the occupation “housewife” and were likely misclassified during data entry. Where possible, such data errors were resolved in consultation with the DMSOs responsible for data collection. However, other errors may have remained undetected.

Second, the method used to classify cases as indigenous or imported in the surveillance system may have caused an overestimation of imported cases. Individuals who travelled outside Zanzibar in the past 30 days could still have contracted malaria locally. Although it is technically possible to genetically determine the approximate origin of a malaria infection using PCR, this approach is not feasible for routine surveillance. Therefore, while the current approach is appropriate, the number of imported cases reported should be interpreted with caution.

Lastly, a considerable number of cases may have been unreported due to the relatively low sensitivity of malaria RDTs (64%) and microscopy (50%) compared to PCR [67]. Some cases may be missed due to undetectably low parasite densities, missed household follow-up visits, and low rates of seeking diagnosis and treatment for malaria at a health facility [12]. Therefore, the increasing malaria incidence in Zanzibar and the scale of the November 2023–March 2024 epidemic warrant serious attention, as the true malaria incidence was likely higher than reported in this study.

## Conclusion

In conclusion, this study characterises the epidemiology of the 2023–2024 malaria epidemic in Zanzibar and demonstrates a notable shift in demographic case profiles, with males aged 15–35 years and living in urban areas representing the highest risk group. Malaria control efforts in Zanzibar must adapt to the changing epidemiology to prevent future large-scale epidemics.

## Supplementary Information

Below is the link to the electronic supplementary material.

Additional file 1.

Additional file 2.

Additional file 3.

## Data Availability

The malaria surveillance datasets analysed in this study are not publicly available because they contain personally identifiable data. However, the aggregated data generated and analysed during this study are included in this published article and its supplementary information files.

## Abbreviations

API: Annual parasite incidence
LLINs: Long-lasting insecticidal nets
IRS: Indoor residual spraying
RDT: Rapid diagnostic tests
ZAHREC: Zanzibar Health Research Ethics Review Committee
ZAHRI: Zanzibar Health Research Institute
ZAMEP: Zanzibar Malaria Elimination Programme
DHIS2: District Health Information Software 2
MCN: Malaria case notification
IR: Incidence rate
TPR: Test positivity rate
RR: Risk ratio
CI: Confidence interval
ITN: Insecticide-treated net
ERA5: ECMWF reanalysis v5
CHIRPS: Climate Hazards Group InfraRed Precipitation with Station data
CCF: Cross-correlation function

## References

[1] Björkman A, Shakely D, Ali AS, Morris U, Mkali H, Abbas AK, et al. From high to low malaria transmission in Zanzibar—challenges and opportunities to achieve elimination. BMC Med. 2019;17:14. 30665398 10.1186/s12916-018-1243-z PMC6341737

[2] Ali MH, Kitau J, Ali AS, Al-Mafazy AW, Tegegne SG, Ussi O, et al. Malaria elimination in Zanzibar: where next? Pan Afr Med J. 2023;45(Suppl 1):7. 37538363 10.11604/pamj.supp.2023.45.1.39804 PMC10395111

[3] Nachilongo H. Zanzibar faces malaria surge despite past successes. The Citizen. 2024. https://www.thecitizen.co.tz/tanzania/zanzibar/zanzibar-faces-malaria-surge-despite-past-successes-4508776. Accessed 2 Feb 2024.

[4] Florescu SA, Larsen CS, Helleberg M, Marin A, Popescu CP, Schlagenhauf P. Upsurge in cases of travellers’ malaria ex Zanzibar indicates that malaria is on the rebound in the archipelago. New Microb New Infect. 2024;57: 101226. 10.1016/j.nmni.2024.101226 PMC10859266 38348216

[5] PMI, ZAMEP, Ifakara Health Institute, Tanzania Communication and Development Center, Population Services International (PSI). 2023 Malaria Surge in Zanzibar Investigation and Response Report. 2023.

[6] WHO. World Malaria Report 2023. Geneva: World Health Organization; 2023. https://www.who.int/teams/global-malaria-programme/reports/world-malaria-report-2023. Accessed 2 Apr 2024.

[7] Institute of Medicine (US) Committee for the Study on Malaria Prevention and Control. Malaria: obstacles and opportunities. Washington: National Academies Press; 1991. 25121285

[8] Lyons CL, Coetzee M, Chown SL. Stable and fluctuating temperature effects on the development rate and survival of two malaria vectors, *Anopheles arabiensis* and *Anopheles funestus*. Parasit Vectors. 2013;6:104. 23590860 10.1186/1756-3305-6-104 PMC3637585

[9] Bisanzio D, Lalji S, Abbas FB, Ali MH, Hassan W, Mkali HR, et al. Spatiotemporal dynamics of malaria in Zanzibar, 2015–2020. BMJ Glob Health. 2023;8: e009566. 36639160 10.1136/bmjgh-2022-009566 PMC9843203

[10] Monroe A, Mihayo K, Okumu F, Finda M, Moore S, Koenker H, et al. Human behaviour and residual malaria transmission in Zanzibar: findings from in-depth interviews and direct observation of community events. Malar J. 2019;18:220. 31262306 10.1186/s12936-019-2855-2 PMC6604484

[11] Le Menach A, Tatem AJ, Cohen JM, Hay SI, Randell H, Patil AP, et al. Travel risk, malaria importation and malaria transmission in Zanzibar. Sci Rep. 2011;1:93. 22355611 10.1038/srep00093 PMC3216579

[12] Das AM, Hetzel MW, Yukich JO, Stuck L, Fakih BS, Al-Mafazy AH, et al. The impact of reactive case detection on malaria transmission in Zanzibar in the presence of human mobility. Epidemics. 2022;41: 100639. 36343496 10.1016/j.epidem.2022.100639 PMC9758615

[13] Mkali HR, Reaves EJ, Lalji SM, Al-Mafazy AW, Joseph JJ, Ali AS, et al. Risk factors associated with malaria infection identified through reactive case detection in Zanzibar, 2012–2019. Malar J. 2021;20:485. 34952596 10.1186/s12936-021-04025-1 PMC8710018

[14] Musiba RM, Tarimo BB, Monroe A, Msaky D, Ngowo H, Mihayo K, et al. Outdoor biting and pyrethroid resistance as potential drivers of persistent malaria transmission in Zanzibar. Malar J. 2022;21:172. 35672768 10.1186/s12936-022-04200-y PMC9171934

[15] The United Republic of Tanzania (URT), Ministry of Finance and Planning, Tanzania National Bureau of Statistics (NBS)and President’s office—finance and planning, office of the chief government statistician (OCGS), Zanzibar. The 2022 Population and Housing Census: Administrative Units Population Distribution Report; Tanzania Zanzibar. 2022. https://sensa.nbs.go.tz/publication/volume1c.pdf Accessed 15 Apr 2024.

[16] Office of the Chief Government Statistician (OCGS). Zanzibar Statistical Abstract 2021. 2022. https://ocgs.go.tz/ReportOCGS/ZANZIBAR%20STATISTICAL%20ABSTRACT%202021.pdf. Accessed 15 Apr 2024.

[17] World Bank Group, OCGS. Towards a More Inclusive Zanzibar Economy : Zanzibar Poverty Assessment 2022 (English). 2022. http://documents.worldbank.org/curated/en/099755011022231706. Accessed 10 Nov 2024.

[18] Ashton RA, Bennett A, Al-Mafazy AW, Abass AK, Msellem MI, McElroy P, et al. Use of routine health information system data to evaluate impact of malaria control interventions in Zanzibar, Tanzania from 2000 to 2015. EClinicalMedicine. 2019;12:11–9. 31388659 10.1016/j.eclinm.2019.05.011 PMC6677660

[19] PMI. U.S. President’s Malaria Initiative Tanzania (Zanzibar) Malaria Operational Plan FY 2024. 2024. https://www.pmi.gov. Accessed 10 Nov 2024.

[20] ZAMEP. National guidelines for malaria surveillance and response in Zanzibar. 2016. https://www.measureevaluation.org/measure-evaluation-tz/resources/national-guidelines-for-malaria-surveillance-and-response-in-zanzibar/at_download/file. Accessed 3 May 2024.

[21] Ministry of Health (MoH) [Tanzania Mainland], Ministry of Health (MoH) [Zanzibar], National Bureau of Statistics (NBS), Office of the Chief Government Statistician (OCGS), ICF. Tanzania demographic and health survey and malaria indicator survey (TDHS-MIS) 2022 final report. Rockville: Maryland; 2023. https://www.dhsprogram.com/pubs/pdf/FR382/FR382.pdf. Accessed 18 Apr 2024.

[22] PMI. FY 2010 Tanzania malaria operational plan. 2011. https://www.pmi.gov. Accessed 10 Nov 2024.

[23] PMI. U.S. President’s Malaria Initiative Tanzania (Zanzibar) Malaria operational plan FY 2023. 2023. www.pmi.gov. Accessed 10 Nov 2024.

[24] Saleh F, Kitau J, Konradsen F, Mboera LEG, Schiøler KL. Assessment of the core and support functions of the integrated disease surveillance and response system in Zanzibar, Tanzania. BMC Public Health. 2021;21: 748. 33865347 10.1186/s12889-021-10758-0 PMC8052932

[25] Van Der Horst T, Al-Mafazy AW, Fakih BS, Stuck L, Ali A, Yukich J, et al. Operational coverage and timeliness of reactive case detection for malaria elimination in Zanzibar, Tanzania. Am J Trop Med Hyg. 2020;102:298–306. 31769395 10.4269/ajtmh.19-0505 PMC7008315

[26] ZAMEP. Malaria surveillance in Zanzibar: data analysis and interpretation. 2016. https://www.measureevaluation.org/measure-evaluation-tz/resources/malaria-surveillance-in-zanzibar-data-analysis-and-interpretation/at_download/file. Accessed 14 Apr 2024.

[27] ZAMEP. Malaria surveillance in Zanzibar field manual for: health facilities, district malaria surveillance officers, and surveillance monitoring and evaluation team. In: Malaria surveillance and operations research. 2016. https://www.measureevaluation.org/measure-evaluation-tz/resources/malaria-surveillance-in-zanzibar-field-manual-for-health-facilities-district-malaria-surveillance-officers-and-surveillance-monitoring-and-evaluation-team/at_download/file. Accessed 14 Apr 2024.

[28] NBS, OCGS. The 2022 population and housing census: age and sex distribution report Tanzania Mainland. 2022. https://sensa.nbs.go.tz/publication/volume2b.pdf. Accessed 14 Apr 2024.

[29] Kulldorff M. A spatial scan statistic. Commun Stat Theory Methods. 1997;2:1481–96.

[30] R Core Team. R: a language and environment for statistical computing. Vienna: R Foundation for Statistical Computing; 2021.

[31] Corp IBM. IBM SPSS statistics for windows. Armonk: IBM Corp; 2021.

[32] QGIS.org. QGIS geographic information system. Bratislava: QGIS Association; 2024.

[33] Hersbach H, Bell B, Berrisford P, Biavati G, Horányi A, Muñoz Sabater J, et al. (2023) ERA5 hourly data on single levels from 1940 to present. Copernicus Climate Change Service (C3S) Climate Data Store (CDS). 2023. 10.24381/cds.adbb2d47

[34] Funk C, Peterson P, Landsfeld M, Pedreros D, Verdin J, Shukla S, et al. The climate hazards infrared precipitation with stations—a new environmental record for monitoring extremes. Sci Data. 2015;2: 150066. 26646728 10.1038/sdata.2015.66 PMC4672685

[35] Cole TJ. Setting number of decimal places for reporting risk ratios: rule of four. BMJ. 2015;350: h1845. 25918351 10.1136/bmj.h1845

[36] Ranjha R, Singh K, Baharia RK, Mohan M, Anvikar AR, Bharti PK. Age-specific malaria vulnerability and transmission reservoir among children. Glob Pediatr. 2023. 10.1016/j.gpeds.2023.100085. 38440360 10.1016/j.gpeds.2023.100085 PMC10911094

[37] Winskill P, Rowland M, Mtove G, Malima RC, Kirby MJ. Malaria risk factors in north-east Tanzania. Malar J. 2011;10:98. 21507217 10.1186/1475-2875-10-98 PMC3094229

[38] Camargo LMA, Ferreira MU, Krieger H, De Camargo EP, Da Silva LP. Unstable hypoendemic malaria in Rondonia (western Amazon region, Brazil): epidemic outbreaks and work-associated incidence in an agro-industrial rural settlement. Am J Trop Med Hyg. 1994;51:16–25. 8059911 10.4269/ajtmh.1994.51.16

[39] Cotter C, Sturrock HJW, Hsiang MS, Liu J, Phillips AA, Hwang J, et al. The changing epidemiology of malaria elimination: new strategies for new challenges. Lancet. 2013;382:900–11. 23594387 10.1016/S0140-6736(13)60310-4 PMC10583787

[40] Chuquiyauri R, Paredes M, Peñataro P, Torres S, Marin S, Tenorio A, et al. Socio-demographics and the development of malaria elimination strategies in the low transmission setting. Acta Trop. 2012;121:292–302. 22100446 10.1016/j.actatropica.2011.11.003 PMC3294046

[41] Khatib B, Mcha J, Pandu Z, Haji M, Hassan M, Ali H, et al. Early evening outdoor biting by malaria-infected *Anopheles arabiensis* vectors threatens malaria elimination efforts in Zanzibar. Malar J. 2025;24:92. 40114159 10.1186/s12936-025-05333-6 PMC11927253

[42] Saleh F, Kitau J, Konradsen F, Kampango A, Abassi R, Schiøler KL. Epidemic risk of arboviral diseases: determining the habitats, spatial-temporal distribution, and abundance of immature *Aedes aegypti* in the urban and rural areas of Zanzibar. Tanzania PLoS Negl Trop Dis. 2020;14: e0008949. 33284806 10.1371/journal.pntd.0008949 PMC7746278

[43] WHO. Global vector control response 2017–2030. Geneva: World Health Organization; 2017. https://www.who.int/publications/i/item/9789241512978. Accessed 3 July 2024.

[44] WHO. Global framework for the response to malaria in urban areas. Geneva: World Health Organization; 2022. https://www.who.int/publications/i/item/9789240061781. Accessed 3 July 2024.

[45] Keiser J, Utzinger J, Caldas De Castro M, Smith TA, Tanner M, Singer BH. Urbanization in sub-Saharan Africa and implications for malaria control. Am J Trop Med Hyg. 2004;71(2):118–27. 15331827

[46] Robert V, Macintyre K, Keating J, Trape JF, Duchemin JB, Warren M, et al. Malaria transmission in urban sub-Saharan Africa. Am J Trop Med Hyg. 2003;68:169–76. 12641407

[47] Hay SI, Guerra CA, Tatem AJ, Atkinson PM, Snow RW. Urbanization, malaria transmission and disease burden in Africa. Nat Rev Microbiol. 2005;3:81–90. 15608702 10.1038/nrmicro1069 PMC3130901

[48] Sattler MA, Mtasiwa D, Kiama M, Premji Z, Tanner M, Killeen GF, et al. Habitat characterization and spatial distribution of Anopheles sp mosquito larvae in Dar es Salaam (Tanzania) during an extended dry period. Malar J. 2005;4:4. 15649333 10.1186/1475-2875-4-4 PMC546229

[49] Awolola TS, Oduola AO, Obansa JB, Chukwurar NJ, Unyimadu JP. *Anopheles gambiae s.s.* breeding in polluted water bodies in urban Lagos, southwestern Nigeria. J Vector Borne Dis. 2007;44:241–4. 18092529

[50] Mattah PAD, Futagbi G, Amekudzi LK, Mattah MM, De Souza DK, Kartey-Attipoe WD, et al. Diversity in breeding sites and distribution of *Anopheles* mosquitoes in selected urban areas of southern Ghana. Parasit Vectors. 2017;10:25. 28086941 10.1186/s13071-016-1941-3 PMC5237286

[51] Walker K, Lynch M. Contributions of *Anopheles* larval control to malaria suppression in tropical Africa: review of achievements and potential. Med Vet Entomol. 2007;21(1):2–21. 17373942 10.1111/j.1365-2915.2007.00674.x

[52] Doumbe-Belisse P, Kopya E, Ngadjeu CS, Sonhafouo-Chiana N, Talipouo A, Djamouko-Djonkam L, et al. Urban malaria in sub-Saharan Africa: dynamic of the vectorial system and the entomological inoculation rate. Malar J. 2021;20:364. 34493280 10.1186/s12936-021-03891-z PMC8424958

[53] Yussuf I. Z’bar needs swift action to control malaria. Daily news. 2024. https://dailynews.co.tz/zbar-needs-swift-action-to-control-malaria/. Accessed 14 July 2024.

[54] Emiru T, Getachew D, Murphy M, Sedda L, Ejigu LA, Bulto MG, et al. Evidence for a role of *Anopheles stephensi* in the spread of drug- and diagnosis-resistant malaria in Africa. Nat Med. 2023;29:3203–11. 37884028 10.1038/s41591-023-02641-9 PMC10719088

[55] Liu Q, Wang M, Du YT, Xie JW, Yin ZG, Cai JH, et al. Possible potential spread of *Anopheles stephensi,* the Asian malaria vector. BMC Infect Dis. 2024;24:333. 38509457 10.1186/s12879-024-09213-3 PMC10953274

[56] Vera-Arias CA, Holzschuh A, Oduma CO, Badu K, Abdul-Hakim M, Yukich J, et al. High-throughput *Plasmodium falciparum hrp2* and *hrp3* gene deletion typing by digital PCR to monitor malaria rapid diagnostic test efficacy. Elife. 2022;11: e72083. 35762586 10.7554/eLife.72083 PMC9246365

[57] Lalji SM, Bisanzio D, Mkali HR, Al Mafazy A-W, Joseph J, Nyinondi S, et al. High-resolution sub-district stratification of malaria risk in Zanzibar. Am J Trop Med Hyg. 2021;105(Suppl 5):210.

[58] Rouamba T, Nakanabo-Diallo S, Derra K, Rouamba E, Kazienga A, Inoue Y, et al. Socioeconomic and environmental factors associated with malaria hotspots in the Nanoro demographic surveillance area, Burkina Faso. BMC Public Health. 2019;19: 249. 30819132 10.1186/s12889-019-6565-z PMC6396465

[59] Kulkarni MA, Duguay C, Ost K. Charting the evidence for climate change impacts on the global spread of malaria and dengue and adaptive responses: a scoping review of reviews. Glob Health. 2022;18:1. 10.1186/s12992-021-00793-2 PMC8725488 34980187

[60] Ouedraogo B, Inoue Y, Kambiré A, Sallah K, Dieng S, Tine R, et al. Spatio-temporal dynamic of malaria in Ouagadougou, Burkina Faso, 2011–2015. Malar J. 2018;17:138. 29609606 10.1186/s12936-018-2280-y PMC5879937

[61] Yamba EI, Fink AH, Badu K, Asare EO, Tompkins AM, Amekudzi LK. Climate drivers of malaria transmission seasonality and their relative importance in sub-Saharan Africa. Geohealth. 2023;7: e2022GH000698. 36743738 10.1029/2022GH000698 PMC9884660

[62] World Meteorological Organization. El Niño weakens but impacts continue. 2024. https://wmo.int/news/media-centre/el-nino-weakens-impacts-continue. Accessed 17 Nov 2024.

[63] IGAD Climate Prediction and Applications Centre (ICPAC). El Niño Southern Oscillation and Indian Ocean Dipole watch for the Eastern Africa—short rains season (October–December 2023). Alerts and early warning. 2023. https://www.icpac.net/news/el-ni%C3%B1o-southern-oscillation-and-indian-ocean-dipole-watch-for-the-eastern-africa-short-rains-season-october-december-2023/. Accessed 19 Nov 2024.

[64] Tanzania meteorological authority (TMA). Statement on the status of Tanzania climate in 2023. 2024. https://www.meteo.go.tz/uploads/publications/sw1718281378-Tanzania%20Climate%20Statements%202023.pdf. Accessed 22 June 2024.

[65] United Republic of Tanzania vice president’s office division of environment. National climate change strategy 2012. 2012. https://faolex.fao.org/docs/pdf/tan191137.pdf. Accessed 22 June 2024.

[66] Cai W, Ng B, Geng T, Jia F, Wu L, Wang G, et al. Anthropogenic impacts on twentieth-century ENSO variability changes. Nat Rev Earth Environ. 2023;4:407–18.

[67] Onken A, Haanshuus CG, Miraji MK, Marijani M, Kibwana KO, Abeid KA, et al. Malaria prevalence and performance of diagnostic tests among patients hospitalized with acute undifferentiated fever in Zanzibar. Malar J. 2022;21:54. 35183188 10.1186/s12936-022-04067-z PMC8858509

