# Supplementary material for: The November-2023–March-2024 malaria epidemic in Zanzibar: a spatiotemporal epidemiological analysis
Source: Malar J. 2025 Oct 22;24:354. doi: 10.1186/s12936-025-05507-2 (PMC12541999; doi:10.1186/s12936-025-05507-2)
Supplement: Supplementary file 3 — Additional file 3. [file 12936_2025_5507_MOESM3_ESM.docx]

# **Supporting information**

**S1 Table.** **Quantitative data sources**

| Data | Data source |
| --- | --- |
| Weekly number of individuals tested for malaria | Integrated Disease Surveillance and Response (DHIS2) |
| Weekly confirmed malaria cases | DHIS2 used only to calculate Test Positivity Rates and Malaria Case Notification (MCN) data for all other incidence rates and analysis. |
| Sex of confirmed malaria cases | MCN |
| Age of those who tested positive for malaria |  |
| Shehia of residence of confirmed malaria cases |  |
| Travel history of confirmed malaria cases |  |
| Long-lasting insecticidal net (LLIN) use among confirmed malaria cases | MCN Household follow-up dataset.  To calculate the risk ratio for insecticide-treated net (ITN) use, this study used LLIN use data collected from confirmed malaria cases during household-follow up visits as a proxy for ITN use. This is due to the absence of data specifically on LLIN use for the general population. LLIN use is expected to reflect ITN use because LLINs are the most commonly distributed and recommended type of ITNs in Zanzibar [1,20] |
| Occupation of confirmed malaria cases |  |
| % of de facto household population who slept under an ITN the night before in the general population | [21] |
| Sex distribution in Zanzibar | Adjusted based on the Intercensal Growth rate of Zanzibar [15]. |
| Population size by Shehia |  |
| Age Distribution per Shehia | Age distribution data is not available at the Shehia level but at the district level [28]. Therefore, the age distribution fractions of the corresponding district were multiplied by the projected 2023 population size to estimate the number of people within that age category within each Shehia. |
| Weekly average temperature | ERA5 hourly data on single levels from 1940 to present [33]. Temperature of air at 2m above the surface. Temporal resolution: Hourly, aggregated into weekly average temperatures. Spatial resolution: 0.25° x 0.25°. |
| Weekly rainfall | Climate Hazards Group InfraRed Precipitation with Station data (CHIRPS). Temporal resolution: Daily, aggregated to weekly total rainfall. Spatial resolution: 0.05° x 0.05°. Built on climatology that incorporates satellite information and station data. [34] |

**S1 Table. ITN use in children under 5 years old in Zanzibar from Jan. 2023 to March 2024**

| Risk factor | | Cases | % | Pop. 2023 | % | Risk (%) | RR (95% CI) |
| --- | --- | --- | --- | --- | --- | --- | --- |
| <5s’ ITN use previous night | Yes | 384 | 45.6 | 186,449 | 65.3 | 0.21 | 1 |
|  | No | 281 | 33.3 | 99,001 | 34.7 | 0.28 | 1.38 (1.18 – 1.61) |
|  | Missing | 178 | 21.1 |  |  |  |  |
| Total | | 843 | 100 | 285,450 | 100 |  |  |

Source: MCN household follow-up data and [21].

**
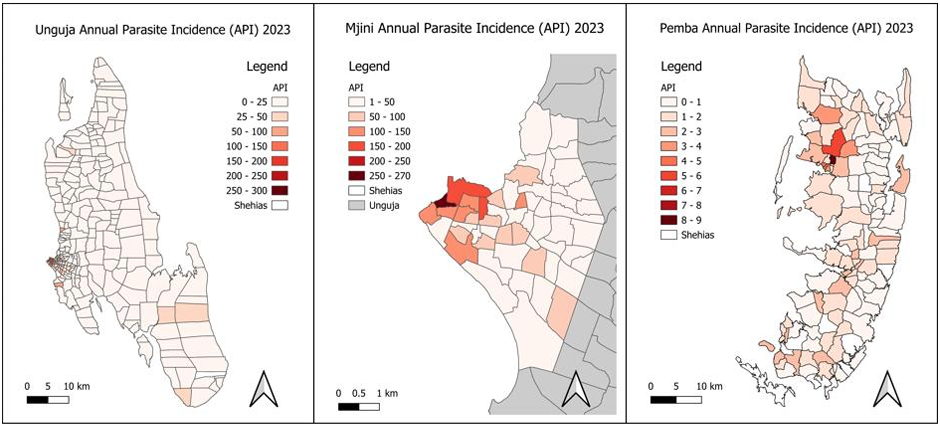
**

**S1 Fig.** 2023 Annual Parasite Incidence in Unguja (A), Mjini (B), and Pemba (C).

**
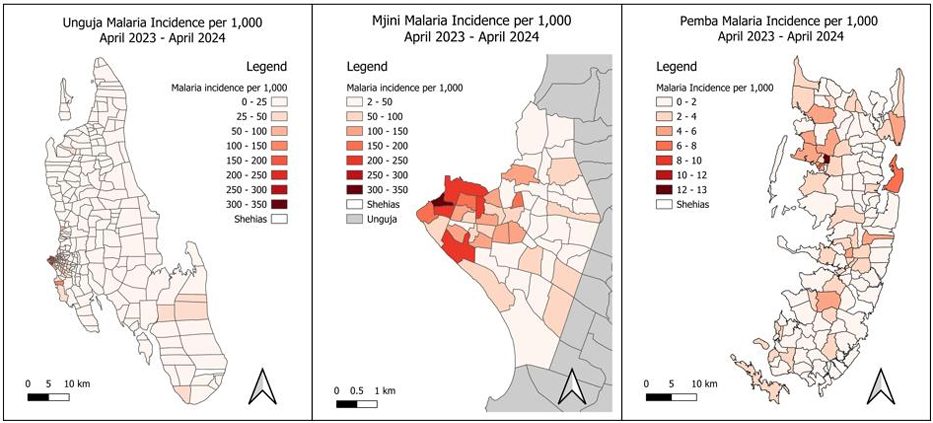
**

**S2 Fig.** Malaria incidence per 1,000 population in Unguja (A), Mjini (B), and Pemba (C) from April 2023 to April 2024.

**
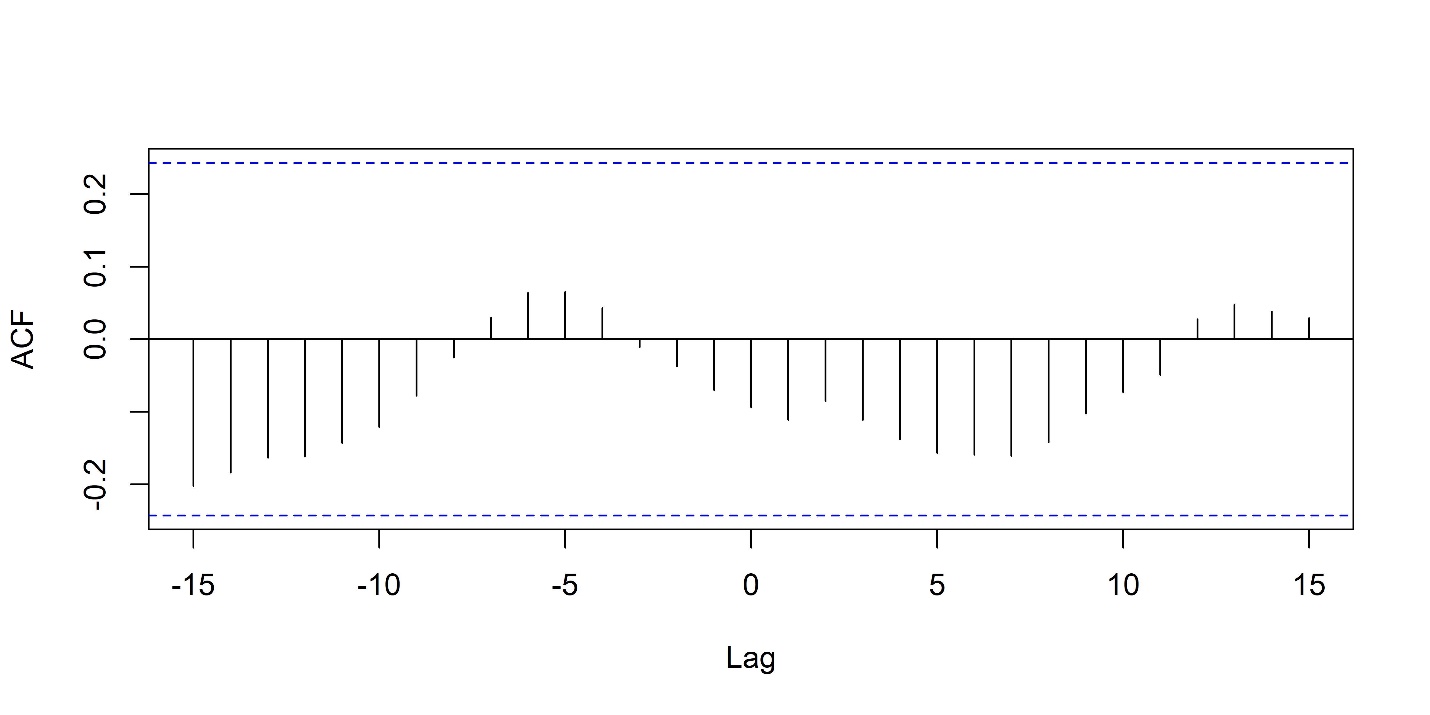
**

**S3 Fig.** Cross-correlation analysis of weekly rainfall and confirmed malaria cases in Zanzibar from Jan. 2023 to March 2024. Vertical black lines crossing the blue dashed line indicate significant correlations (p<0.05).

**
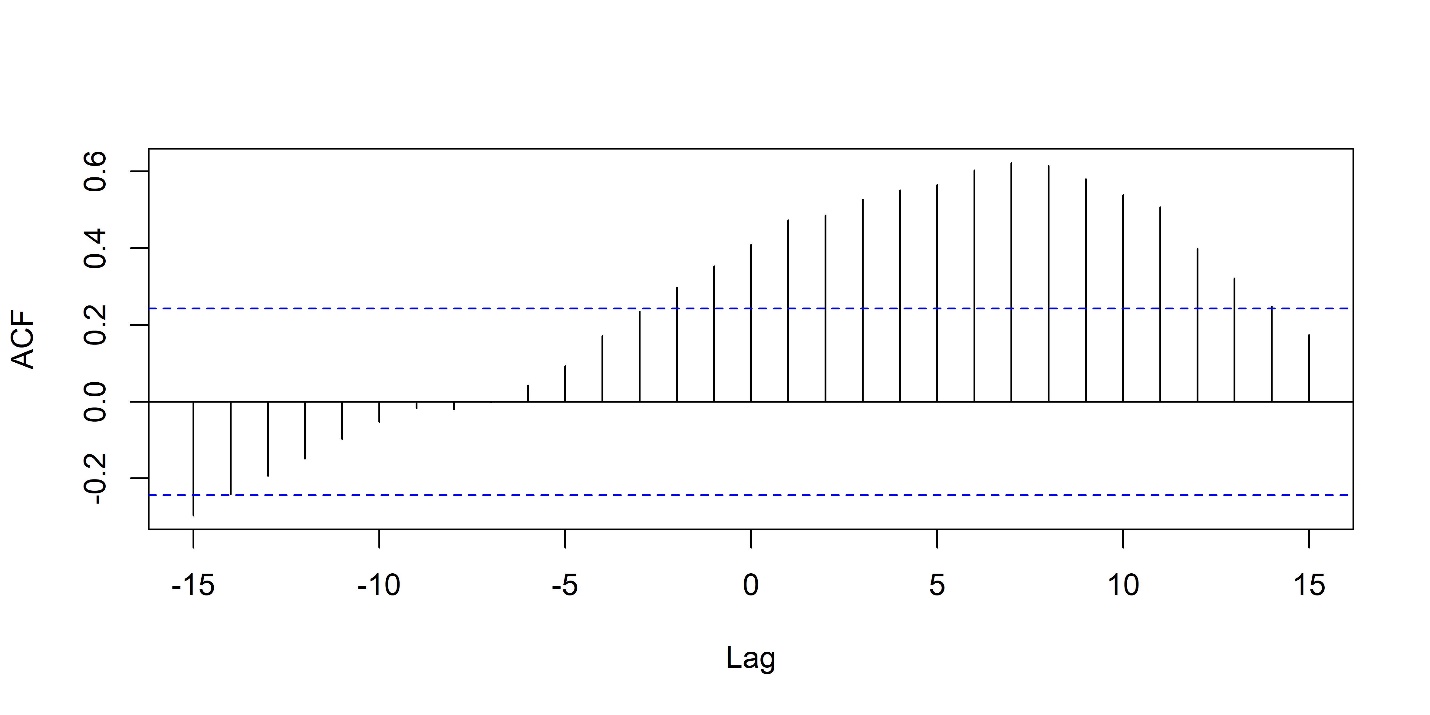
**

**S4 Fig.** Cross-correlation analysis of weekly average temperature and confirmed malaria cases in Zanzibar from Jan. 2023 to March 2024. Vertical black lines crossing the blue dashed line indicate significant correlations (p<0.05).
